# Leaf and root litter decomposition is discontinued at high altitude tropical montane rainforests contributing to carbon sequestration

**DOI:** 10.1002/ece3.3189

**Published:** 2017-07-10

**Authors:** Franca Marian, Dorothee Sandmann, Valentyna Krashevska, Mark Maraun, Stefan Scheu

**Affiliations:** ^1^ J.F. Blumenbach Institute of Zoology and Anthropology University of Göttingen Göttingen Germany; ^2^ Centre of Biodiversity and Sustainable Land Use University of Göttingen Göttingen Germany

**Keywords:** altitudinal gradient, Ecuador, litter quality, litter type, litterbag, microbial biomass

## Abstract

We investigated how altitude affects the decomposition of leaf and root litter in the Andean tropical montane rainforest of southern Ecuador, that is, through changes in the litter quality between altitudes or other site‐specific differences in microenvironmental conditions. Leaf litter from three abundant tree species and roots of different diameter from sites at 1,000, 2,000, and 3,000 m were placed in litterbags and incubated for 6, 12, 24, 36, and 48 months. Environmental conditions at the three altitudes and the sampling time were the main factors driving litter decomposition, while origin, and therefore quality of the litter, was of minor importance. At 2,000 and 3,000 m decomposition of litter declined for 12 months reaching a limit value of ~50% of initial and not decomposing further for about 24 months. After 36 months, decomposition commenced at low rates resulting in an average of 37.9% and 44.4% of initial remaining after 48 months. In contrast, at 1,000 m decomposition continued for 48 months until only 10.9% of the initial litter mass remained. Changes in decomposition rates were paralleled by changes in microorganisms with microbial biomass decreasing after 24 months at 2,000 and 3,000 m, while varying little at 1,000 m. The results show that, irrespective of litter origin (1,000, 2,000, 3,000 m) and type (leaves, roots), unfavorable microenvironmental conditions at high altitudes inhibit decomposition processes resulting in the sequestration of carbon in thick organic layers.

## 1 INTRODUCTION

Decomposition is among the most fundamental processes in terrestrial ecosystems with up to 99% of the aboveground net primary production entering the decomposer food web as leaf and root litter (McNaughton et al., 1989). It is therefore particularly important to know how decomposition processes are regulated and this aspect is attracting increasing attention in the current debate on how ecosystems function as carbon (C) sinks or sources (Cox et al., 2013; Todd‐Brown et al., 2014). Immense amounts of C are stored in dead organic matter, with peatlands, tundra, and the boreal zone containing an estimated one‐third of the global C stock (Gorham, 1991; Post et al., 1982). Release of this C contributes substantially to increasing atmospheric CO_2_ concentrations (Cox et al., 2013). There are several current models that attempt to predict the response of soil organic C pools to climate change or to elevated atmospheric CO_2_ (Cox et al., 2013). None of them, however, indicate clearly whether C storage will increase or decline. This uncertainty calls for more data on the factors regulating terrestrial C storage (Cox et al., 2013; Todd‐Brown et al., 2014). The main factors involved are litter quality, climate, and the composition of the decomposer community (Berg, 2014; Coûteaux et al., 2002; Davidson & Janssens, 2006; Kirschbaum, 1995). Litter quality is known to regulate decomposition processes mainly in the early stages of decomposition, while climatic conditions are more important in later stages (Berg & McClaugherty, 2008). The sensitivity of decomposition processes to changes in temperature have been widely studied but with contradictory outcomes (Bruijnzeel & Veneklaas, 1998; Davidson & Janssens, 2006; Fang et al., 2005; Fierer et al., 2005; Gholz et al., 2000; Giardina & Ryan, 2000; Kirschbaum, 1995; Knorr et al., 2005). The effects on litter decomposition of both litter quality and temperature are mediated through their effects on the microbial community (Allison, Wallenstein, & Bradford, 2010; Cleveland et al., 2014; Treseder et al., 2012). Therefore, to understand the variations caused by litter quality and temperature, it is necessary to investigate changes in microbial characteristics.

Large stocks of dead organic material are not only stored in peatlands of the boreal zone, but also in tropical regions (Bruijnzeel & Veneklaas, 1998; Pan et al., 2011; Post et al., 1982; Tanner, Vitousek, & Cuevas, 1998). Decomposition is generally faster in the humid tropics than in temperate regions (Heneghan et al., 1999). At high altitude, however, litter decomposition in tropical montane rainforests is slower than in lowland tropical rainforests. Organic C therefore accumulates in the high altitude forests (Bruijnzeel & Veneklaas, 1998; Butenschoen et al., 2014; Dieleman et al., 2013; Heneghan et al., 1999; Post et al., 1982; Tanner et al., 1998). In the tropical Andes, one of the most species‐rich and diverse ecosystems on earth (Barthlott et al., 2005; Henderson, Churchill, & Luteyn, 1991; Hilt & Fiedler, 2005), montane rainforests are exposed to strong variation in biotic and abiotic conditions on small spatial scales (Homeier et al., 2010). The higher the altitude the slower the litter decomposes and so soil organic matter and soil C stocks increase (Illig et al., 2008; Leuschner et al., 2007; Wilcke et al., 2002). These altitudinal variations are associated with changes in plant community composition (Homeier et al., 2010; Moser, Hertel, & Leuschner, 2007; Paulsch, Piechowski, & Müller‐Hohenstein, 2006; Wilcke et al., 2008) and declining quality of leaf litter material with increasing altitude, resulting in the formation of soil organic matter layers of low quality at high altitude (Wilcke et al., 2002). The biomass of living and dead fine roots as well as that of living coarse roots increases significantly with increasing altitude (Girardin et al., 2010; Leuschner et al., 2007). Therefore, fine root necromass is likely to contribute significantly to the formation of thick organic layers at high altitudes in tropical montane forest ecosystems. In addition, because plant community composition changes with altitude, the plant‐associated mycorrhiza community changes in the same way (Kottke & Haug, 2004; Kottke et al., 2006). These mycorrhizal communities are generally dominated, in tropical montane ecosystems, by arbuscular mycorrhizal fungi (AMF) (Kottke et al., 2004). It has recently been suggested that the mycorrhizal fungal communities strongly influence decomposition processes and soil C stocks (Averill, Turner, & Finzi, 2014). Temperature and precipitation also change with altitude (Moser et al., 2007; Röderstein, Hertel, & Leuschner, 2005), and Wilcke et al. (2002) suggested that low temperatures, increased precipitation and water logging are responsible for the accumulation of soil C in high altitude Andean forest ecosystems. The same has been suggested for other tropical forests (Bruijnzeel & Veneklaas, 1998; Tanner et al., 1998). In contrast with this view, Krashevska et al. (2012) reported that microbial biomass decreases with declining precipitation and Illig et al. (2008) found temperature to only be of minor importance for decomposition processes in montane Andean forest ecosystems. This suggests that other factors in addition to moisture and temperature are crucial for the regulation of litter decomposition in tropical montane forest ecosystems.

To clarify these effects, we investigated how altitude affects the decomposition of leaf and root litter in the Andean tropical montane rainforest of southern Ecuador, that is, through changes in litter quality or other site‐specific differences in microenvironmental conditions. We measured C and nitrogen (N) as well as microbial biomass concentrations in a litterbag experiment including both leaf and root litter exposed in the field for 4 years. We expected that (1) decomposition rates as well as changes in C and N concentration in decomposing litter material are largely driven by the quality of the litter material, that is the origin of the litter from rainforests of different altitudes, and that (2) decomposition of root litter is slower than that of leaf litter with the difference varying with the origin of the litter material. We also expected that (3) variations in microbial biomass and ergosterol concentration in litter are closely linked to changes in the litter decomposition processes. Overall, the study aims at improving the understanding of the mechanisms contributing to the accumulation of dead organic material in high altitude tropical rainforests.

## 2 MATERIALS AND METHODS

### 2.1 Study site

The study area was in southern Ecuador on the eastern slope of the Andes. Within this area we established three sites along an altitudinal gradient. The sites were at 1,000, 2,000, and 3,000 m a.s.l. and in the northern part of the Podocarpus National Park facing northeast to northwest. The slopes are moderately steep at 26–31° (Moser et al., 2007) and covered with montane rainforest that is largely undisturbed (Homeier, Dalitz, & Breckle, 2002). The site at 1000 m (Bombuscaro, S04°06′54″, W78°58′02″) is south of the city Zamora in the valley of the Rio Bombuscaro. The site at 2,000 m (San Francisco, S3°58′18″, W79°4′45″) is part of the Reserva Biologica San Francisco at the northern border of the Podocarpus National Park. The site at 3,000 m (Cajanuma, S04°06′711″, W79°10′58″) is south of the city Loja at the northwest gate of the Podocarpus National Park.

With 8–10 humid months per year the region has a semihumid climate. Mean annual rainfall is little different at the 1,000 and 2,000 m sites (2,230 and 1,950 mm, respectively), but much higher at the 3,000 m sites (4,500 mm; Moser et al., 2007). The mean annual air temperature is negatively related to altitude being 19.4, 15.7, and 9.4°C at 1,000, 2,000 and 3,000 m, respectively. Soil pH is similarly related to altitude being 3.94, 3.52, and 2.86 at 1,000, 2,000, and 3,000 m, respectively (Moser et al., 2007). Mean soil moisture in the organic layer increases with increasing altitude being 9.9, 11.6, and 45.3 vol. % at 1,000, 2,000, and 3,000 m, respectively (Leuschner et al., 2007). Biotic conditions also change along the altitudinal gradient. Mean tree height decreases with altitude being 15.6, 10.1, and 5.2 m at 1,000, 2,000, and 3,000 m, while the thickness of the organic layer, the fine root biomass (within the top 0.30 m), and the necromass (within the top 0.2 m) increases (Graefe, Hertel, & Leuschner, 2008; Moser et al., 2007; Röderstein et al., 2005). The thickness of the organic layer at the three sites increases steeply with altitude, being 48, 305, and 435 mm at 1,000, 2,000, and 3,000 m, respectively. The biomass of fine roots has the same pattern with respective values of 2.7, 6.2, and 10.8 t ha^−1^ (Graefe et al., 2008). The soil types at 1,000 m are predominantly Alumic Acrisols, at 2,000 m Gley Cambisols and at 3,000 m Podzols (Moser et al., 2007). At 1,000 m the litter layer overlies mineral soil (Ah horizon), that is, there are no F or H layers. In contrast, at 2,000 and 3,000 m the leaf litter overlies thick organic layers predominantly comprised of F‐material. At each of the three altitudes the density of macrofauna, such as earthworms, diplopods and isopods, in the litter and upper soil layer is low compared to that in temperate forests (Illig et al., 2008; Maraun et al., 2008).

### 2.2 Experimental setup

Nylon bags (litterbags, 4 mm mesh) were used to investigate the decomposition and microbial colonization of leaves and roots, that is, two types of litter materials. The mesh size allows access to the litter by the dominant decomposer mesofauna but minimizes the loss of litter due to handling. Leaf litter was created for a site by collecting freshly fallen leaves of the three most abundant tree families at that site (origins; Bombuscaro = Bomb, Estacion Scientifica San Francisco = ECSF, Cajanuma = Caja). Root litter was obtained by digging up the upper 0.20–0.30 m of organic material and soil, and picking out the roots by hand. The roots were then cleaned of adhering organic matter and soil by rinsing them gently with tap water. These roots were then sorted into three size classes, small (<2 mm diameter), medium (2–5 mm diameter) and large (>5 mm diameter). Both leaf litter and roots where dried at 60°C for 4 days. The quality of the litter (as assessed by C‐to‐N ratio and percentages of C and N) differed markedly between leaf litter species and root size classes and generally declined with altitude. The decline was particularly clear in roots (Table 1).

**Table 1:** Leaf litter species (left) and root litter size classes (right) which were placed into the litterbags, their respective mean C‐to‐N ratio (C/N), percentage of C and N and the amount of each used (g dry weight) from the three study sites Bombuscaro (Bomb, 1,000 m), Estacion Cientifica San Francisco (ECSF, 2,000 m), and Cajanuma (Caja, 3,000 m). The amount of leaf litter of each species and the quantity of roots from each size class placed into the litterbags of the three litter species was based on the amounts present at each study site

Leaves					Roots				
Bomb	g	C/N	C %	N %	Bomb	g	C/N	C %	N %
*Puteria* sp.	5	41.4	50.7	1.2	Small	2.8	42.3	51.3	1.2
*Cavendishia* sp.	3	48.1	49.8	1.0	Medium	4.9	45.9	51.2	1.1
*Mollinedia* sp.	2	22.8	49.2	2.2	Large	2.5	57.8	47.8	0.8
ECSF					ECSF				
*Graffenrieda emarginata*	5	76.5	49.2	0.6	Small	4.4	53.0	50.1	0.9
*Clusia* sp.	4	94.5	51.4	0.6	Medium	2.1	73.9	49.8	0.7
*Cavendishia* sp.	1	72.5	50.1	0.7	Large	3.5	88.4	49.5	0.6
Caja					Caja				
*Clusia* sp.	5	104.1	51.6	0.5	Small	3.4	63.7	52.0	0.8
*Graffenrieda emarginata*	4	75.1	50.2	0.7	Medium	2.5	101.4	51.9	0.5
*Hediosmum* sp.	1	39.4	46.9	1.2	Large	4.1	185.5	51.2	0.3

**Figure 1:**
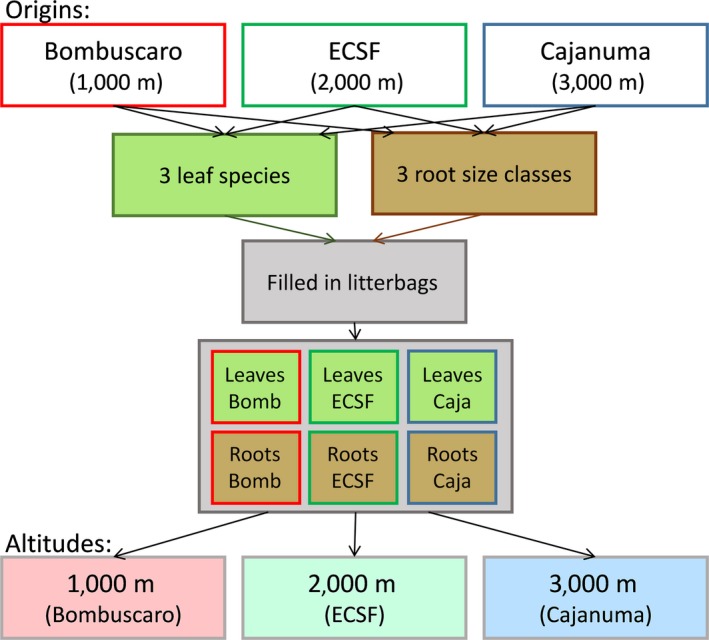
Scheme of collection of litter materials (origins) and placement of litterbags in the field (altitudes). Leaf and root litter samples from three sites [origins; Bombuscaro (Bomb, 1,000 m), Estacion Scientifica San Francisco (ECSF, 2,000 m) and Cajanuma (Caja, 3,000 m)] were placed in litterbags; six litterbags, three with leaves and three with roots, were set up per site and placed in the field at each of the respective sites where leaf litter and roots were sampled

Leaves of the three plant families or roots of the three root size classes were mixed according to their relative abundances at each altitude. Litterbags were then filled with 10 g of leaf litter or of roots (Table 1). Litterbags filled with the six different litter mixtures (three origins × two types), were placed at each site (Figure 1). Leaf litterbags were placed on top of the litter layer, whereas root litterbags were placed horizontally at the interface between the top of the mineral soil (1,000 m) or between the F‐layer (2,000 m and 3,000 m) and the litter (L) layer where fine root density is at a maximum (Röderstein et al., 2005). Four blocks were established at each of the three study sites with a minimum distance between blocks of 20 m. Five replicates of each treatment were placed in each block with one replicate from each block being retrieved at each of five dates, that is, after 6, 12, 24, 36, and 48 months. There were thus 360 litterbags in total.

### 2.3 Analytical procedures

After retrieval, the remaining leaf and root litter were cleaned by removing roots that had grown into the litterbags. The litter was then dried at 60°C for 4 days and its dry mass measured gravimetrically. For measuring C and N concentrations, an aliquot of the litter material from each bag was separately milled to a powder and tin capsules were filled with 3–4 mg from each sample. C and N in the powder were then analyzed using an elemental analyser (Vario EL III, elementar, Hanau, Germany).

Microbial respiration and substrate‐induced respiration (SIR) were determined by measuring O_2_ consumption using a computer‐controlled O_2_ microcompensation apparatus (Scheu, 1992). For these measurements, material from each litterbag was homogenized by cutting it into pieces of about 0.5 cm^2^ and the water content was adjusted to about 60% of the water holding capacity. Before measuring, the samples were rested for 4 days at room temperature to avoid including in the measurements the increased basal respiration caused by the homogenization. Then, O_2_ consumption was measured for 24 hr. Microbial biomass was determined by measuring substrate‐induced respiration (Anderson & Domsch, 1978). Moist samples equivalent to 0.2 g dry weight were supplemented with glucose equivalent to 80 mg g^−1^ dry weight and O_2_ consumption measured for a further 24 hr. Microbial biomass was calculated from the maximum initial respiratory response (MIRR; μl O_2_ g^−1^ dry mass h^−1^) as Cmic = 38 × MIRR (Beck et al., 1997; Joergensen & Scheu, 1999).

Ergosterol was extracted from 0.5 g of each sample of leaf or root material. Each 0.5 g was placed with 50 ml ethanol in a widemouthed amber glass jar and the jars then agitated on an oscillating shaker for 30 min (250 rev. min^−1^). The samples were then centrifuged at 3,500 g for 40 min. The supernatant was split in two 20 ml samples and each evaporated separately. The dried extract was collected in 0.5 ml methanol and filtered through a membrane of 0.45 μm. Ergosterol was determined by reverse‐phase high‐performance liquid chromatography (HPCL) (Djajakirana, Joergensen, & Meyer, 1996). Ergosterol concentrations were only measured from the first three sampling dates (6, 12, and 24 months) due to lack of material at later dates, resulting in a total of 216 samples.

### 2.4 Calculations and statistical analysis

We focused on the variation in the amount and concentration of C within the litter material in order to be able to link decomposition processes closely to energetic processes. Therefore, the amounts of C remaining (C_R_) in the litterbags at the sampling dates (*n*) were expressed as percentages of the initial amount of C placed into the litterbags (C_0_). Similarly, changes in the amount of N remaining (N_R_) were expressed as percentage of the initial amount of N placed in the litterbags (N_0_), according to the following formulas:

C_R_ [%] = (C_n_/C_0_) × 100 and N_R_ [%] = (N_n_/N_0_) × 100, with C_n_ and N_n_ the amount of C and N remaining at each sampling date *n*.

The concentrations of C (C_C_) and N (N_C_) in the litter were calculated as:

C_C_ [%] = (C_n_/DW_n_) × 100 and N_C_ [%] = (N_n_/DW_n_) × 100, with DW_n_ the dry weight of litter remaining at sampling date *n*.

In addition, the litter C‐to‐N mass ratio was calculated but, as variations were similar to those in N_C_ (Tables S2 and S3 in Appendix S1), we focused on N_C_ and display C‐to‐N ratio in the appendix (Table S1 and Fig. S1 in Appendix S1).

Microbial activity and density were quantified using four parameters: microbial biomass (C_mic_), metabolic oxygen quotient (qO_2_), the ratio of microbial carbon to total soil carbon (C_mic_‐to‐C_org_ ratio), and the ergosterol concentration. However, as qO_2_ and C_mic_‐to‐C_org_ ratio and ergosterol concentration responded in a similar way to C_mic_, they are only displayed in the appendix (Fig. S2a–c and Table S1 in Appendix S1).

The remaining amount and concentration of C and N (C_R_, N_R_, C_C_, N_C,_ and C‐to‐N ratio) and microbial parameters (C_mic_, qO_2_, C_mic_‐to‐C_org_ ratio, and ergosterol concentration) were analyzed by repeated measures four‐factor randomized complete block multivariate analysis of variance (MANOVA) with time (6, 12, 24, 36, and 48 months) as repeated factor and block (1, 2, 3, and 4) being nested for location. Fixed factors were altitude (1,000, 2,000 and 3,000 m a.s.l.), origin (Bomb, ECSF, and Caja) and litter type (leaf and root litter). Block was excluded from the analysis as there were no significant block effects. Protected repeated measures analysis of variance (ANOVA) with the same criteria as stated above were carried out with the general linear model (GLM; type III sum of squares), providing between and within subject effects and significant variation between sampling dates (Scheiner & Gurevitch, 2001). Single ANOVAs for each sampling date were performed to identify differences between dates. Tukey's HSD test (*p* < .05) was used to identify significant differences between means, especially between sampling dates. Statistical analyses were performed using SAS (Statistical Analysis System, Version 9.3; SAS Institute Inc., Cary, NC, USA).

All the parameters measured were correlated in a matrix and for each sampling date separately in order to test for collinearity using STATISTICA 12 (Statsoft, Inc., Tulsa, USA). Before the analyses, data were inspected for homogeneity of variance and normal distribution. Percentage data were arcsine square‐root‐transformed and all other data log‐transformed. Data of different transformation type were not analyzed in the same MANOVA. Means presented in the Results are based on nontransformed data.

## 3 RESULTS

Altitude had the strongest influence on N_R_, C_C_, qO_2_ and C_mic_‐to‐C_org_ ratio. Litter type had the strongest influence on the ergosterol concentration, while C_R_ and C_mic_ varied most strongly with sampling date. The origin of litter material had the strongest influence on N_C_ (Table 2). The effect of the origin of litter material on most of the parameters investigated was only significant at early sampling dates, affecting C_mic_ and ergosterol concentration after 6 months, C_mic_‐to‐C_org_ ratio after 6 and 24 months, and C_C_ after 12 and 36 months. C_R_ and qO_2_ were not affected by the origin of the litter under main effects at any sampling date. Only N_R_ and N_C_ significantly varied with litter origin at all of the sampling dates. The following results therefore focus on the effects of altitude, sampling date, and litter type.

**Table 2:** Repeated measures ANOVA/GLM table of *F*‐ and *p*‐values on the effects of altitude (1,000, 2,000, and 3,000 m), litter type (roots or leaves), litter origin [Bombuscaro (Bomb), ECSF and Cajanuma (Caja)], and date (6, 12, 24, 36, and 48 months) on the amount of C (C_R_) and N (N_R_) in litterbags (as percentages of initial), the percentage of C (C_C_) and N (N_C_) in the litter as well as the microbial biomass (C_mic_). Significant effects are given in bold

	C_R_		C_C_		N_R_		N_C_		C_mic_	
	*F*‐value	*p*‐value	*F*‐value	*p*‐value	*F*‐value	*p*‐value	*F*‐value	*p*‐value	*F*‐value	*p*‐value
Between subject effects										
Altitude	**219.26**	**<.0001**	**111.59**	**<.0001**	**103.48**	**<.0001**	**45.66**	**<.0001**	**15.08**	**<.0001**
Origin	1.18	.3163	**5.76**	**.0057**	**35.81**	**<.0001**	**337.75**	**<.0001**	**8.77**	**.0007**
Type	**22.38**	**<.0001**	**36.89**	**<.0001**	3.86	.0552	**128.82**	**<.0001**	0.02	.8868
Altitude × origin	1.64	.1790	**6.54**	**.0003**	2.52	.0527	**3.45**	**.0147**	2.04	.1083
Altitude × type	0.39	.6819	**47.53**	**<.0001**	**3.67**	**.0326**	2.82	.0696	**9.11**	**.0006**
Origin × type	**7.91**	**.0011**	0.13	.8761	**7.00**	**.0021**	**9.23**	**.0004**	0.37	.6937
Altitude × origin × type	**2.59**	**.0482**	2.50	.0548	**3.02**	**.0263**	**3.75**	**.0098**	1.70	.1694
Within subject effects										
Date	**271.93**	**<.0001**	**58.88**	**<.0001**	**78.99**	**<.0001**	**136.79**	**<.0001**	**22.63**	**<.0001**
Date × altitude	**48.78**	**<.0001**	**16.19**	**<.0001**	**30.55**	**<.0001**	**5.73**	**<.0001**	**8.16**	**<.0001**
Date × origin	0.58	.7933	**2.63**	**.0093**	1.80	.0791	**6.02**	**<.0001**	1.94	.0581
Date × type	**5.80**	**.0002**	1.00	.4093	**5.20**	**.0005**	**3.96**	**.0041**	**2.98**	**.0210**
Date × altitude × origin	1.13	.3322	1.52	.0943	**1.91**	**.0216**	**2.68**	**.0008**	0.85	.6314
Date × altitude × type	0.44	.8983	**7.11**	**<.0001**	0.30	.9665	**4.70**	**<.0001**	**6.31**	**<.0001**
Date × origin × type	1.43	.1876	1.96	.0541	**2.62**	**.0095**	**4.03**	**.0002**	0.83	.5745
Date × altitude × origin × type	0.61	.8715	1.51	.0994	0.51	.9409	0.62	.8633	1.51	.1033

### 3.1 Amount and concentration of C and N

**Figure 2:**
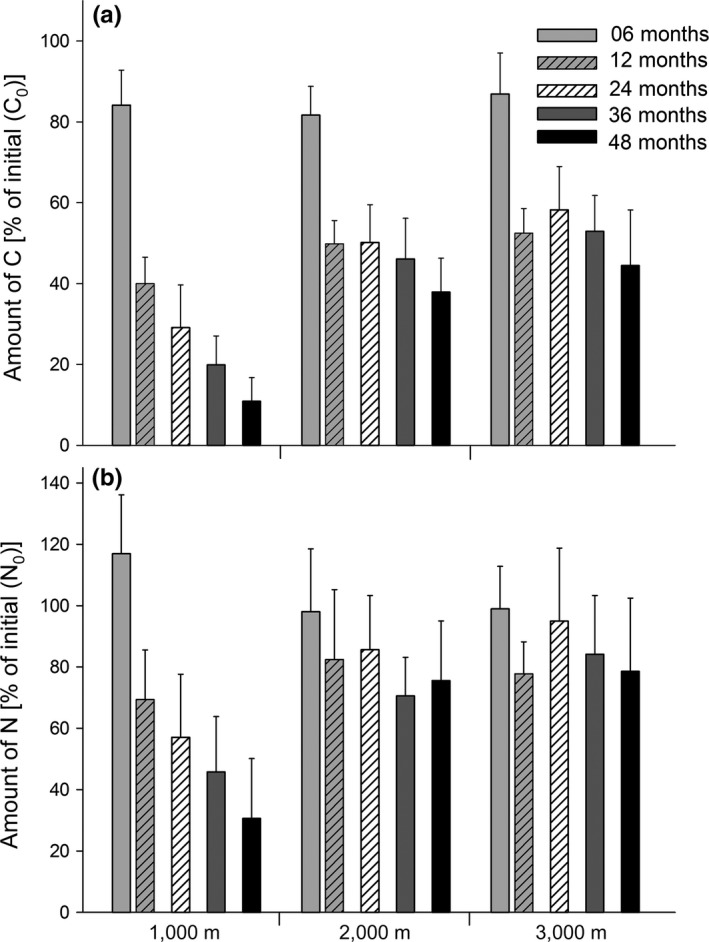
Variation in the amount of C (C_R_) (a) and N (N_R_) (b) in litter material (pooled from three origins) (percentages of initial) exposed in tropical montane rainforests at three altitudes (1,000, 2,000, and 3,000 m) for 6, 12, 24, 36, and 48 months (means ± *SD*)

**Figure 3:**
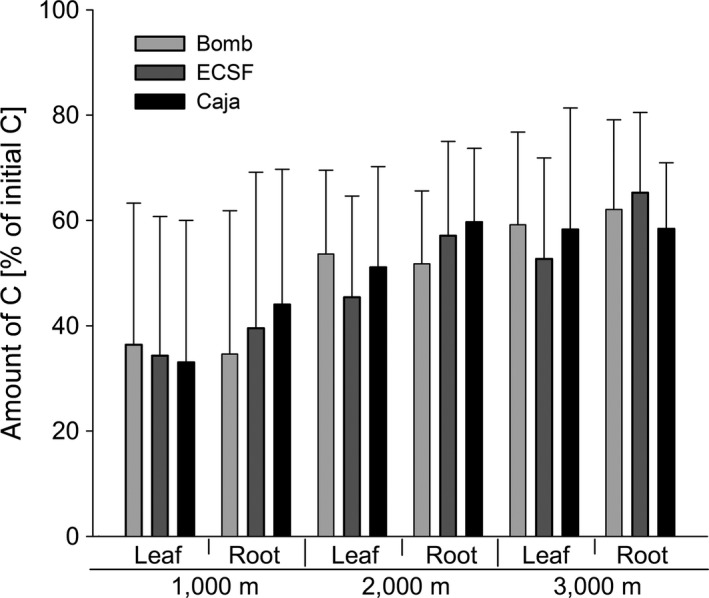
Variation in the amount of C remaining (C_R_; percentages of initial) in leaf and root litter from three altitudes (Bomb, ECSF, and Caja) exposed in tropical montane rainforests at three altitudes (1,000, 2,000, and 3,000 m), pooled over five sampling dates (6, 12, 24, 38, and 48 months) (means ± *SD*)

C_R_ declined with time but this varied significantly with altitude (Figure 2a, Table 2). Within the first 6 months the reduction in C_R_ at each of the three altitudes was similar and averaged 15.8 ± 2.6% of C_0_. In contrast, from 6 to 12 months the reduction in C_R_ was greatest at 1,000 m (44.1% of C_0_), but similar at 2,000 and 3,000 m (at 31.9 and 34.4% of C_0_). From 12 to 36 months, C_R_ remained almost constant at the two highest sites with an average of 48.7 ± 2.2 at 2,000 and of 54.5 ± 3.2% of C_0_ at 3,000 m. From 36 to 48 months, C_R_ again decreased, by 8.2 of C_0_ at 2,000 and 8.5% at 3,000 m. In contrast, at 1,000 m C_R_ decreased steadily from 12 to 48 months by an average of 9.7 ± 1.1% of C_0_ each year resulting in only 10.9% of C_0_ remaining after 48 months. In contrast, much more remained at the two highest sites, 37.9% of C_0_ at 2,000 and 44.4% at 3,000 m. At 1,000 m, C_R_ differed significantly between sampling dates (Tukey's HSD test; *p* < .05). In contrast, at 2,000 and 3,000 m C_R_ did not differ significantly between 12, 24, and 36 months, but C_R_ at these dates differed significantly from that at 6 and 48 months (Tukey's HSD test; *p* < .05). The reduction in C_R_ also differed between root and leaf litter (significant date × type interaction; Table 2). Within the first year, C_R_ decreased to a similar extent in leaf and root litter, with the reductions averaging 15.8 ± 0.5% of C_0_ after 6 months and 36.9 ± 1.6% of C_0_ from 6 to 12 months. Between 12 and 24 months the reduction in C_R_ slightly differed between leaf and root litter. In leaf litter, the reduction was 5.0% of C_0_ reaching 41.7% of C_0_ but the amount in root litter remained almost constant at 49.1 ± 1.3% of C_0_. From 24 to 48 months the reduction was similar, averaging 6.4 ± 0.7% from 24 to 36 and 8.1 ± 0.9% of C_0_ from 36 to 48 months. This produced a lower overall decrease of C_R_ by 48 months in root litter (35.5% of C_0_) compared to leaf litter (26.8% of C_0_). Furthermore, C_R_ also varied with the origin of the litter material but this depended on altitude and litter type (Figure 3, Table 2). At each of the three altitudes, C_R_ in root litter from ECSF was higher than that of leaf litter from ECSF, while in litter from Caja this was only true at 1,000 and 2,000 m. C_R_ in litter from Bomb did not vary significantly between leaf and root litter.

**Figure 4:**
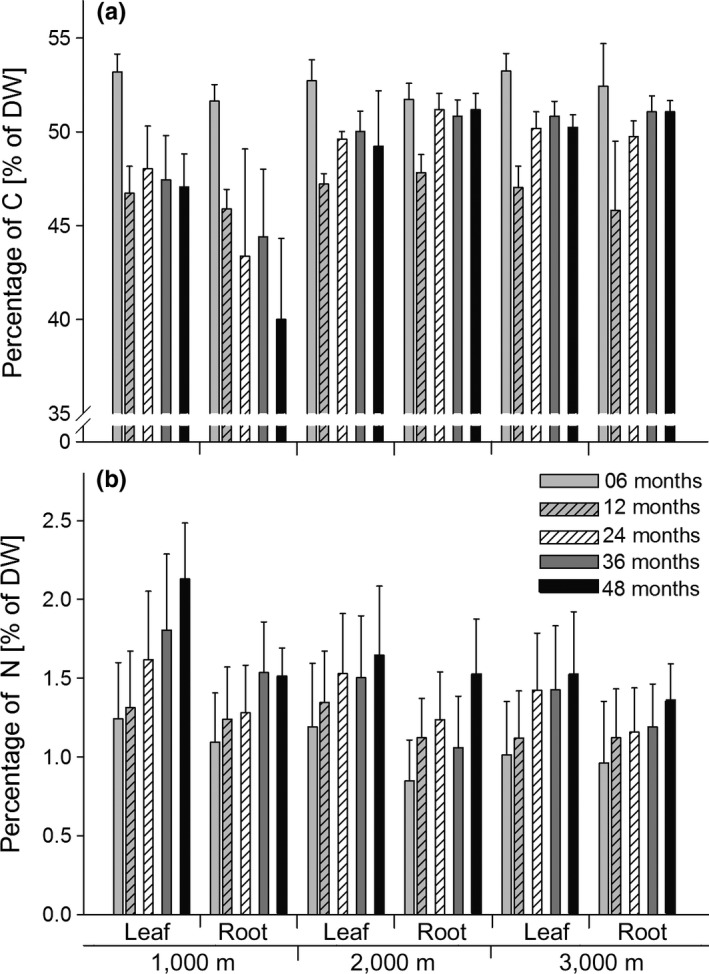
Variation in the percentages (of dry weight) of C (C_C_) (a) and N (N_C_) (b) in leaf and root litter exposed in tropical montane rainforests at three altitudes (1,000, 2,000, and 3,000 m) for 6, 12, 24, 36, and 48 months (means ± *SD*)

The initial C_C_ of the litter placed in the litterbags was generally high but differed between leaf litter (50.1, 50.2, and 50.6% for leaf litter from Bomb, ECSF, and Caja, respectively), and root litter (respective values of 50.4, 49.8, and 51.6%). During decomposition, C_C_ varied significantly with time, litter type, and altitude (Table 2, Figure 4a). However, it decreased at each of the altitudes in both litter types from 6 to 12 months. The pattern differed in subsequent years. In both leaf and root litter exposed at 2,000 and 3,000 m C_C_ increased from 12 to 24 months and then remained almost constant in the following 2 years. At 1,000 m variations in C_C_ with time differed between leaf and root litter. In leaf litter, C_C_ remained almost constant after 12 months at an average of 47.3 ± 0.6%. However, in root litter, it slowly decreased but remained almost constant between 24 and 36 months. Overall, C_C_ of leaf litter was slightly higher than that of root litter but it was generally lower in leaf litter at 1000 m because of the greater reduction with time at this site. In addition, C_C_ also varied with time and the origin of the litter (Table 2). This was mainly due to lower C_C_ in the litter material from ECSF after 36 months. After 6 months, C_C_ in litter from Bomb was slightly lower (51.8%) than in litter from ECSF and Caja (both 52.8%). The decrease from 6 to 12 months was the greatest and did not differ with litter origin decreasing, on average, to 46.8%. The C_C_ in litter from Bomb and Caja varied similarly. It increased by 1.2% in both places after a year and by 2.5% in both places after 2 years. In the third year, the increase was 1.2% for litter from Bomb and 0.9% for litter from Caja. In the fourth year, C_C_ decreased in both places by an average of 1.4 ± 0.1% per year. The C_C_ in litter from ECSF only increased by 2.0% from 12 to 24 months. From 24 to 36, it decreased by 1.0% and then stayed constant from 36 to 48 months. In consequence, litter from Bomb and ECSF had slightly lower C_C_ after 48 months (47.9 ± 4.9 and 48.0 ± 4.3% C_C_, respectively) than litter from Caja (48.7 ± 4.3%).

Except during the first 6 months, N_R_ generally decreased with time but this differed significantly with altitude (Figure 2b, Table 2). After the first 6 months, N_R_ exceeded N_0_ at 1000 m, whereas it uniformly decreased later. The reduction was greatest from 6 to 12 months when the amount fell by 47.5% of N_0_. The decline continued in subsequent years at an average of 12.9 ± 2.0% of N_0_ per year. At 2,000 and 3,000 m the pattern was less consistent. At both altitudes N_R_ was close to 100% of N_0_ after 6 months and decreased in the following 6 months to 82.4% of N_0_ at 2,000 and 77.7% at 3,000 m. From 12 to 24 months N_R_ remained almost constant at 2,000 m reaching only 85.6% after 24 months, but at 3,000 m N_R_ increased to 94.9% of N_0_. From 24 to 36 months it decreased again to 70.6% of N_0_ at 2,000 and 84.1% at 3,000 m. From 36 to 48 months, changes were small. The reduction in N_R_ also differed significantly between litter types (Table 2). In both leaf and root litter, the decline was greatest between 6 and 12 months. In leaf litter, N_R_ remained constant between 12 and 24 months but decreased between 24 and 48 months. In root litter, N_R_ fluctuated after 12 months with an overall decline lower than that in leaf litter. Root litter lost only 30.8% of N_0_ after 48 months but leaf litter lost 45.5%. The reduction in N_R_ at the three altitudes also differed significantly with the origin of the litter material (Table 2). The reduction was most pronounced at 1,000 m, with only 17.0% of N_0_ in litter from Bomb after 48 months compared to 26.9% of N_0_ in litter from Bomb and 46.2% in litter Caja. At higher altitudes, the decrease was also at a maximum in litter from Bomb with 57.2% and 73.1% of N_0_ after 48 months at 2,000 and 3,000 m, respectively, as compared to 88.3% and 81.2% of N_0_ in litter from ECSF and Caja at 2,000 m, respectively, and 76.9 and 86.5% of N_0_ in litter from ECSF and Caja at 3,000 m, respectively. The reduction in N_R_ in the two litter types also differed significantly with the origin of the litter (Table 2). N_R_ declined most strongly in leaf litter from Bomb with 45.1% of N_0_ left after 48 months compared to 62.4% of N_0_ from ECSF leaf litter and 55.9% from Caja leaf litter. In root litter from Bomb and ECSF, the N loss was very similar with an average of 56.4% of N_0_ from Bomb and 64.6% from ECSF after 48 months as compared to 86.7% of N_0_ in root litter from Caja.

Initial N_C_ of the litter materials in the litterbags was generally low but differed among sites for leaf litter (1.35, 0.62, and 0.64% for leaf litter from Bomb, ECSF, and Caja, respectively) and root litter (1.09, 0.76, and 0.53% for leaf litter from Bomb, ECSF, and Caja, respectively). During decomposition, N_C_ generally increased, but the increase was low and differed with altitude and litter type (Figure 4b, Table 2). The increase was greatest in leaf litter at 1,000 m which increased by 0.9% between 6 and 48 months and in root litter at 2,000 m which increased by 0.7% between 6 and 48 months (Figure 4b). In the other treatments, the increase in N_C_ varied between 0.4 and 0.5% over the course of 48 months. In leaf litter at 2,000 and 3,000 m, the increase ceased between 24 and 36 months, while in root litter at 3,000 m it ceased between 12 and 36 months. In contrast, N_C_ in root litter at 2,000 m decreased in the period of 24 to 36 months. N_C_ increased strongly in leaf litter at 1,000 m, while it increased less in root litter, remaining constant between 12 and 24 months as well as between 36 and 48 months. Overall, N_C_ decreased with increasing altitude and was higher in leaf litter than in root litter. In addition to altitude and litter type the changes in N_C_ during decomposition also differed with litter origin (Table 2). Litter from Bomb, which had the highest initial N_C_, had the lowest increase irrespective of altitude. At 1,000 and 3,000 m, the increase in N_C_ was most pronounced in litter from Caja while at 2,000 m it was most pronounced in litter from ECSF. In litter from Bomb and Caja, N_C_ decreased or remained constant between 24 and 36 months at both 2,000 and 3,000 m. Generally, the increase in N_C_ between 6 and 48 months in the litter from Bomb and ECSF was lower in root (0.4 and 0.6%, respectively) than in leaf litter (0.6 and 0.7%, respectively). In contrast, in litter from Caja the increase in N_C_ in root litter (0.6%) exceeded that in leaf litter (0.5%).

### 3.2 Microorganisms

**Figure 5:**
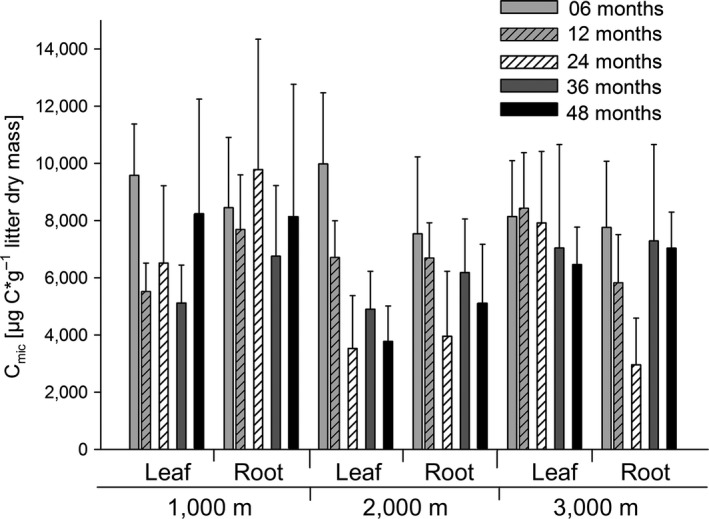
Variation in microbial biomass (C_mic_) in leaf and root litter material (pooled from three origins) exposed in tropical montane rainforests at three altitudes (1,000, 2,000, and 3,000 m) for 6, 12, 24, 36, and 48 months (means ± *SD*)

C_mic_ varied significantly with time, altitude, and litter type (Figure 5, Table 2). It declined strongly from 6 to 24 months in both litter types at 2,000 m and in root litter at 3,000 m, followed by an increase from 24 to 36 months. In leaf litter at 3,000 m, C_mic_ remained almost constant. In leaf litter at 1,000 m, C_mic_ declined from 6 to 12 months, remained constant until 36 months and strongly increased from 36 to 48 months. In contrast, in root litter at 1,000 m, C_mic_ increased strongly after 24 months followed by a similarly strong decrease. C_mic_ also varied with the origin of the litter material (Table 2). It was highest in litter from Bomb followed by litter from ECSF and litter from Caja. Changes in C_mic_‐to‐C_org_ ratio, qO_2_ and ergosterol concentration closely correlated with, and resembled, changes in C_mic_ (see Tables S2 and S3, Fig. S2 in Appendix S1).

## 4 DISCUSSION

Decomposition processes depend strongly on the quality of litter material, especially in the early phase of decomposition (Berg & McClaugherty, 2008; Cusack et al., 2009). In contrast to this view, we showed that altitude affects decomposition by modifying microenvironmental conditions rather than the quality of the litter, that is, litter origin, which only slightly influenced the amount of C remaining (C_R_) in the early phase of decomposition.

Decomposition processes follow different stages with the early stage being characterized by rapid mass loss via the leaching of soluble compounds (Berg, 2014; Berg & McClaugherty, 2008). Our results support this view, with a rapid decline in the amount of litter C within the first year of decomposition irrespective of altitude. Berg (2014) described that in the later phases of decomposition the rate of mass and C loss slows down and is dominated by the degradation of the remaining recalcitrant litter compounds such as lignin. Klotzbücher et al. (2011), however, showed that to a large extent lignin is also degraded early during decomposition. The decomposition rate can even cease in later stages, reaching a limit value and leaving behind recalcitrant litter compounds which are little decomposed (Berg, 2014; Berg & McClaugherty, 2008). We discovered that the reduction in C_R_ in later phases (from 12 months onwards) varied between altitudes. Butenschoen et al. (2014), in a litterbag experiment at our 2,000 m study site, reported a great reduction of litter mass in the first year after which decomposition slowed down or stopped from 12 to 24 months. Our results support these findings and document that the retardation in decomposition rate and the decrease in C_R_ at 2,000 and 3,000 m starts after 12 months and then lasts for at least 24 months, with the amount of litter C staying constant at a limit value of about 50% of C_0_. After this second phase of litter decomposition, the loss of C resumed at 2,000 and 3,000 m but at a low rate of 8–9% of C_0_ between 36 and 48 months. This resumption of C loss indicates a third phase of decomposition which probably is associated with slow degradation of recalcitrant litter compounds such as lignin and a shift in the decomposer community toward lignin decomposing microorganisms. The long delay of this resumption of decomposition suggests that the late microbial community, degrading recalcitrant litter compounds, only establishes through facilitation by the activity of the early colonisers and the degradation of labile litter compounds. In contrast, at 1,000 m C_R_ continued to decrease after 12 months at a low but steady rate of around 10% of C_0_ each year, reaching <20% C_R_ after 48 months. This pattern indicates that, in contrast to higher altitudes, higher nutrient availability at 1,000 m allows continuous decomposition of litter including recalcitrant compounds and this results in lower accumulation of litter material. In temperate forests decomposition dynamics and humus formation are strongly influenced by the composition of the decomposer community (Eisenhauer et al., 2007; Ponge, 2003; Salmon, Artuso, Frizzera, & Zampedri, 2008). Similar to the pattern at our 1,000 m site, a thin litter layer overlying an Ah horizon (mull humus) in temperate regions is associated with abundant macrofauna. In contrast, at all the three altitudes the density of macrofauna in the litter and upper soil layer is low compared to temperate forests (Illig et al., 2008; Maraun et al., 2008). Macrofauna density, therefore, cannot explain the difference in decomposition rates between the study sites. Differences in climatic conditions, that is, precipitation and temperature, and soil characteristics, that is, soil pH and soil moisture, also influence decomposition patterns (Aerts, 1997; Fierer et al., 2005; Scheu et al., 2003; Wieder, Cleveland, & Townsend, 2009). However, the sensitivity to variations in these parameters, especially temperature, differs between ecosystems, climatic zones and between different qualities of the litter material (Aerts, 1997; Blagodatskaya et al., 2016; Fierer et al., 2005). We showed in our study that decomposition patterns were similar at the two higher altitudes although temperature, precipitation, soil moisture, and soil pH varied between each of the three study sites. The strong increase in precipitation and reduction in air temperature from 2,000 to 3,000 m did not, however, translate into strong differences in litter decomposition. Illig et al. (2008) also found temperature to be of minor importance for decomposition processes along a shorter gradient at the same study site as ours. Further, our study shows that precipitation and soil pH also are of little importance. We presume that the different forest floor types at the different altitudes contribute to the different decomposition dynamics. At 1,000 m the litter layer is in close contact with mineral soil, whereas thick layers of F‐material separate leaf litter and mineral soil at higher altitudes. This conclusion is supported by the fact that the concentration of C in the litter material (C_C_), reflecting the concentration of condensed C compounds (Berg & McClaugherty, 2008), remained high over the course of the experiment, in particular at 2,000 and 3,000 m. At 1,000 m, however, it declined markedly, particularly in root litter.

Soil characteristics influence decomposition, for example by supporting a stable microbial community that acquires N from the inorganic N pool in soil (Hodge, Robinson, & Fitter, 2000). At 1,000 m the thin litter layer on top of the mineral soil enables direct interactions between microorganisms of the litter and mineral soil while, at the higher altitude sites, the litter layer is separated from the mineral soil by thick layers of F‐material with low N concentrations. This separation may hamper nutrient capture and translocation into the L‐layer by fungi that facilitate litter decomposition (Lummer, Scheu, & Butenschoen, 2012). The lack of N transfer into the litter may cause a feedback loop, that is, the accumulation of organic material and the formation of thick F layers further inhibiting litter decomposition. The retardation in the decrease of C_R_ after 12 months was generally more pronounced in root litter, while in leaf litter C mass loss continued at low rates. We presume that this is because root litter contains higher concentrations of lignin and lower concentrations of N than leaf litter, resulting in a greater accumulation of recalcitrant litter compounds in the later phases of decomposition. This is supported by the fact that the thick F‐layers at 2,000 and 3,000 m are formed largely by root litter that is little decomposed (Leuschner et al., 2007).

The initial N_C_ of both leaf and root litter materials was generally low and increased during exposure at all the altitudes and in both litter types with the changes in N_C_ varying with the initial N_C_. In litter material with initially high N_C_, the increase with time was less pronounced than in litter material with low N_C_, suggesting that N_C_ values converge with time. Despite the low initial N_C_ in the litter materials, there was generally no increase in the amount of N in the litter material (N_R_) except within the first 6 months in litter at 1,000 m, indicating that only at this site fungal hyphae transported N into the litter. This contrasts decomposition of litter materials low in N in temperate and boreal forest ecosystems which typically accumulate N for longer periods of time (Berg, 2014). After 6 months N_R_ uniformly decreased suggesting that N from litter was mobilized and transported out of the litterbags. The continuous loss of C at 1000 m, together with N_R_ decreasing from 12 months onwards, suggests that recalcitrant compounds were also decomposed. The reduction in N_R_ at later stages of litter decay also varied with the initial N_C_. In litter material with low initial N_C_, the increase in N_C_ was stronger than in litter material with high initial N_C_. At 2,000 and 3,000 m N_R_ decreased by less than 30% of N_0_, and this decrease mainly occurred between 6 and 12 months of exposure in the field, suggesting that during this early phase export of litter N was at a maximum, while at later phases of decomposition litter N was retained in the litter. N mineralization overall is low at the three study sites investigated and decreases with increasing altitude (Baldos, Corre, & Veldkamp, 2015; Martinson, Corre, & Veldkamp, 2013). The C‐to‐N ratio of litter at 2,000 and 3,000 m was high and ranged between 97 and 32 during the experiment (see Fig. S1 in Appendix S1), values at which net mineralization is assumed to be low or nonexisting (Hodge et al., 2000). This suggests that plant roots at the study site are unable to obtain a sufficient amount of N from decomposing litter. Plants presumably rely on mycorrhizal fungi improving N capture by growing into leaf and root litter material. Indeed, the great majority of tree species at our study sites are associated with AM fungi (Haug et al., 2004; Kottke et al., 2004). AM fungi, while unable to decompose complex organic molecules themselves, stimulate N uptake by plants and improve decomposition by interacting with the microbial community (Coleman, 1994; Hodge, Campbell & Fitter, 2001; Koller Rodriguez, et al., 2013). The microbial community presumably relies heavily on mycorrhizal C provided via hyphal exudates, in particular at 2,000 and 3,000 m (Bonkowski, 2004; Koller, Scheu, et al., 2013). This C input potentially enables the microbial community to obtain N from the litter material despite the high C‐to‐N ratio (Koller, Robin, et al., 2013). In fact, irrespective of litter C‐to‐N ratios, protozoa mobilize microbially fixed N by grazing on bacteria and AM fungi translocate the mobilized N to the host plant (Koller, Rodriguez, et al., 2013). Notably, Krashevska et al. (2008) suggested that testate amoebae, a major group of protists at our study site, are driven by the availability of bacteria and fungi as food sources.

The structure and functioning of the microbial community in decomposing litter material changes during decomposition in parallel to changes in the chemical composition of the litter material (Berg & McClaugherty, 2008; Scheu & Parkinson, 1995). This linkage likewise was evident throughout the decomposition process in our study. While the origin of the litter material affected the microbial community mainly early during decomposition, site‐specific conditions became more important later. At all altitudes, the rapid decrease in C_R_ and N_R_ from 6 to 12 months, as well as the generally high C_mic_ after 6 months, suggest that the early microbial community depends mainly on labile litter compounds. This is also reflected by high C_mic_ in litter material from Bomb which had the lowest C‐to‐N ratio. The ergosterol concentration decreased from 6 to 12 months in leaf litter at 1,000 and 2,000 m, indicating that saprotrophic fungi also depended on labile litter compounds (see Fig. S2 in Appendix S1). C_mic_ was generally low compared to temperate regions, resembling results from previous studies at our study sites and other tropical forests (Imberger & Chiu, 2002; Krashevska et al., 2008; Luizao, Bonde, & Rosswall, 1992). This again supports our conclusion that litter resources are of low quality and difficult to decompose due to low N and high concentrations of recalcitrant compounds.

Close linkage between C mass loss and C_mic_ was particularly evident after 12 months. At 1,000 m, C_mic_ was generally higher than at 2,000 and 3,000 m, resembling the patterns in C mass loss. Continuous decomposition of litter at 1,000 m suggests that microorganisms at this altitude can continuously decompose recalcitrant litter compounds including lignin thereby reducing C_C_ and C_R_. This probably is facilitated by the close contact of litter and mineral soil at 1,000 m (see above). In contrast, separation of the leaf litter layer from mineral soil and low N concentration of F‐material at 2,000 and 3,000 m presumably hamper translocation of N into the litter causing the litter to remain poor in N. In fact, both leaf and root litter was still poor in N after 48 months with average C‐to‐N ratios of 33.7 ± 9.0 at 2,000 and 36.7 ± 8.2 at 3,000 m. At 1,000 m, however, litter N was higher with an average C‐to‐N ratio of 24.8 ± 4.7 after 48 months. Even after 48 months, litter C‐to‐N ratios at 2,000 and 3,000 m therefore were considerably above the threshold at which microorganisms are assumed to be able to mobilize N (Hodge et al., 2000). This again supports our conclusion that net N mobilization from the litter was due to trophic interactions between saprotrophic microorganisms, microbial grazers and VA mycorrhizal fungi (see above).

In both leaf and root litter at 2,000 m and root litter at 3,000 m, the strong decrease in C_mic_ from 6 to 24 months indicates that the early microbial community depending on easily decomposable litter compounds declined due to substrate depletion. High qO_2_ during this phase (see Fig. S2 in Appendix S1) suggests that increased nutrient stress contributes to the decline in C_mic_ (Blagodatskaya & Anderson, 1999). After 24 months C_mic_ increased while qO_2_ decreased, indicating a shift in the structure of the microbial community. This suggests that after depletion of easily decomposable litter compounds in the first phase of decomposition, the activity of the microbial community slowed down or ceased in the second phase between 12 and 36 months. Resumption of litter decomposition at 2,000 and 3,000 m in the third phase after 36 months suggests that a novel microbial community able to decompose condensed recalcitrant litter compounds including lignin took over. Slower decline in C_mic_ and higher ergosterol concentration in leaf litter at 3,000 m compared to 2,000 m indicates that this shift in microbial community composition was less strong in leaf litter at 3,000 m compared to root litter and both litter types at 2,000 m. The dominance of saprotrophic fungi in the upper litter layer presumably is most pronounced at 3,000 m where organic layers are deepest reaching a thickness of more than 0.4 m (Graefe et al., 2008).

## 5 CONCLUSIONS

The results of our long term study indicate that the accumulation of dead organic material at high altitude and the formation of thick F‐layers in tropical montane rainforests is, in part caused by reduced or halted decomposition of both leaf and root litter in the second phase of decomposition between 12 and 36 months as well as slow resumption of litter decomposition after this phase. Continuous decomposition of all litter types at 1,000 m suggests that altitude did not affect decomposition through differences in litter quality but through site‐specific conditions such as temperature, precipitation, different forest floor types, and different trophic interactions between plants and the belowground community. We suggest that the accumulation of leaf and root litter at high altitudes and therefore the formation of thick layers of organic material (F‐layer) inhibit positive interactions between the microbial community in the upper litter layer and the mineral soil. This causes the microbial community to depend only on plant‐derived resources, with the litter poor in N being insufficient to allow saprotrophic microorganisms to effectively decompose recalcitrant litter compounds such as lignin. We suggest that these conditions lead to closer trophic linkage between plants, microorganisms and microbial grazers at higher altitude allowing VA mycorrhizal fungi to capture N locked up in litter even though the litter C‐to‐N ratio exceeds the threshold where N typically becomes available for plant uptake. Future studies need to elucidate interactions between the plant and the decomposer community in order to disentangle how litter and root‐derived resources affect belowground community structure, decomposition processes and the capture of nutrients from decomposing litter by plants.

## AUTHOR CONTRIBUTIONS

Franca Marian involved in investigation, formal analysis, and writing the original draft. Dorothee Sandmann and Valentyna Krashevska involved in investigation; formal analysis; and writing, review, and editing of the manuscript. Mark Maraun and Stefan Scheu involved in conceptualization; methodology; writing, review, and editing of the manuscript; supervision; and funding acquisition. We acknowledge support by the German Research Foundation and the Open Access Publication Funds of the Göttingen University.

## CONFLICT OF INTEREST

None declared.
