# Supplementary material for: Leaf and root litter decomposition is discontinued at high altitude tropical montane rainforests contributing to carbon sequestration
Source: Ecol Evol. 2017 Jul 10;7(16):6432–43. doi: 10.1002/ece3.3189 (PMC5574766; doi:10.1002/ece3.3189)
Supplement: Supplementary file 1 [file ECE3-7-6432-s001.docx]

**Appendix S1**

**Article title:** Leaf and root litter decomposition is discontinued at high altitude tropical montane rainforests contributing to carbon sequestration

**Authors:** Franca Marian, Dorothee Sandmann, Valentyna Krashevska, Mark Maraun, Stefan Scheu

**Table S1:** Repeated measured ANOVA/GLM table of F- and p-values on the effects of altitude (1000, 2000 and 3000 m), litter type (roots or leaves), litter origin (Bombuscaro (Bomb), ECSF and Cajanuma (Caja)) and sampling date (after 6, 12, 24, 36 and 48 months) on the C-to-N ratio, metabolic oxygen quotient (qO_2_), C_mic_‑to‑C_org_ ratio and ergosterol content (ergosterol/C_org_). Significant effects are given in bold.

|  | **C-to-N ratio** | | **qO2** | | **C_mic_-to-C_org_ ratio** | | **ergosterol** | |
| --- | --- | --- | --- | --- | --- | --- | --- | --- |
|  | **F-value** | **p-value** | **F-value** | **p-value** | **F-value** | **p-value** | **F-value** | **p-value** |
| ***Between subject effects*** |  |  |  |  |  |  |  |  |
| **location** | **89.39** | **<.0001** | **16.2** | **<.0001** | **16.01** | **<.0001** | **13.55** | **<.0001** |
| **origin** | **348.71** | **<.0001** | 2.17 | 0.1299 | **11.18** | **0.0002** | 2.71 | 0.0762 |
| **type** | **89.28** | **<.0001** | 0.01 | 0.9248 | 0.40 | 0.5307 | **90.31** | **<.0001** |
| **location × origin** | **3.45** | **0.0148** | **3.43** | **0.0185** | **3.65** | **0.0135** | **4.21** | **0.0051** |
| **location × type** | **8.61** | **0.0006** | 1.16 | 0.3241 | **11.11** | **0.0002** | **12.67** | **<.0001** |
| **origin × type** | **8.38** | **0.0008** | 2.62 | 0.0872 | 0.66 | 0.5227 | **8.48** | **0.0007** |
| **location × origin × type** | **4.70** | **0.0028** | **2.77** | **0.0429** | 1.46 | 0.234 | 1.64 | 0.1785 |
|  |  |  |  |  |  |  |  |  |
| ***Within subject effects*** |  |  |  |  |  |  |  |  |
| **date** | **189.98** | **<.0001** | **12.33** | **<.0001** | **12.9** | **<.0001** | **19.96** | **<.0001** |
| **date × location** | **10.09** | **<.0001** | **3.36** | **0.0015** | **7.55** | **<.0001** | **13.61** | **<.0001** |
| **date × origin** | **6.49** | **<.0001** | 0.79 | 0.6128 | **2.08** | **0.0419** | **5.19** | **0.0008** |
| **date × type** | **3.79** | **0.0055** | 1.58 | 0.1825 | 2.4 | 0.0525 | **13.23** | **<.0001** |
| **date × location × origin** | **2.09** | **0.0102** | 1.41 | 0.1455 | 1.06 | 0.3984 | 1.31 | 0.2484 |
| **date × location × type** | **2.89** | **0.0046** | **5.54** | **<.0001** | **4.92** | **<.0001** | **3.45** | **0.011** |
| **date × origin × type** | **3.48** | **0.0009** | 0.91 | 0.5102 | 0.51 | 0.8482 | 1.87 | 0.1208 |
| **date × location × origin × type** | 0.56 | 0.9089 | 1.07 | 0.3942 | 0.97 | 0.4958 | 0.55 | 0.8139 |

**Fig. S1:** Variation in C‑to‑N ratio in leaf and root litter exposed in tropical montane rainforests at three altitudes (1000, 2000 and 3000 m) for 6, 12, 24, 36 and 48 months.

Fig.S2: Variation in metabolic oxygen quotient (qO_2_) (a), Cmic‑to‑Corg ratio (b) and ergosterol concentration (c) in leaf and root litter exposed in tropical montane rainforests at three altitudes (1000, 2000 and 3000 m) for 6, 12, 24, 36 and 48 months.

**Table S2:** Correlation matrix between the amount of C (C_R_) and N (N_R_), the percentage of C (C_C_) and N (N_C_), C‑to‑N ratio (C/N), microbial biomass (C_mic_), C_mic_‑to-C_org_ ratio (C_mic_/C_org_) and metabolic oxygen quotient (qO_2_) in decomposing litter material exposed in tropical montane rainforests for 6, 12, 24, 36 and 48 months.

|  | **C_R_** | **N_R_** | **C_C_** | **N_C_** | **C/N** | **C_mic_** | **C_mic_/C_org_** |
| --- | --- | --- | --- | --- | --- | --- | --- |
| **N_R_** | **p=0.003** |  |  |  |  |  |  |
| **C_C_** | **p<0.000** | **p=0.002** |  |  |  |  |  |
| **N_C_** | **p<0.001** | p=0.123 | **p<0.001** |  |  |  |  |
| **C/N** | **p<0.001** | **p<0.001** | **p<0.001** | **p=0.040** |  |  |  |
| **C_mic_** | **p<0.001** | **p<0.001** | **p<0.001** | **p=0.001** | **p=0.002** |  |  |
| **C_mic_/C_org_** | p=0.382 | **p<0.001** | p=0.118 | p=0.066 | **p<0.001** | **p<0.001** |  |
| **qO_2_** | **p<0.001** | **p<0.001** | **p<0.001** | **p=0.002** | **p<0.001** | p=0.942 | p=0.371 |

Table S3: Correlation matrix between the amount of C (C_R_) and N (N_R_), the percentage of C (C_C_) and N (N_C_), C‑to‑N ratio (C/N), microbial biomass (C_mic_), C_mic_‑to-C_org_ ratio (C_mic_/C_org_), metabolic oxygen quotient (qO_2_) and the ergosterol content, after exposure in tropical montane rainforests for 6 (a), 12 (b), 24 (c), 36 (d) and 48 months (e).

| (a) | **C_R_** | **N_R_** | **C_C_** | **N_C_** | **C/N** | **C_mic_** | **C_mic_/C_org_** | **qO_2_** |
| --- | --- | --- | --- | --- | --- | --- | --- | --- |
| **N_R_** | p=0.077 |  |  |  |  |  |  |  |
| **C_C_** | p=0.062 | p=0.377 |  |  |  |  |  |  |
| **N_C_** | p=0.212 | p=0.238 | **p=0.001** |  |  |  |  |  |
| **C/N** | p=0.074 | **p<0.001** | p=0.435 | **p=0.039** |  |  |  |  |
| **C_mic_** | p=0.536 | **p<0.001** | p=0.317 | p=0.554 | **p<0.001** |  |  |  |
| **C_mic_/C_org_** | p=0.181 | **p<0.001** | p=0.255 | p=0.643 | **p<0.001** | **p<0.001** |  |  |
| **qO_2_** | p=0.259 | p=0.075 | **p=0.003** | p=0.292 | p=0.097 | **p=0.046** | **p=0.037** |  |
| **Ergosterol** | **p=0.021** | p=0.840 | p=0.189 | p=0.249 | p=0.686 | **p<0.001** | **p<0.001** | p=0.134 |
|  |  |  |  |  |  |  |  |  |
| (b) | **C_R_** | **N_R_** | **C_C_** | **N_C_** | **C/N** | **C_mic_** | **C_mic_/C_org_** | **qO_2_** |
| **N_R_** | p=0.734 |  |  |  |  |  |  |  |
| **C_C_** | **p=0.006** | p=0.886 |  |  |  |  |  |  |
| **N_C_** | p=0.292 | p=0.285 | **p<0.001** |  |  |  |  |  |
| **C/N** | p=0.408 | **p<0.001** | p=0.534 | p=0.280 |  |  |  |  |
| **C_mic_** | p=0.872 | p=0.136 | p=0.342 | p=0.361 | p=0.219 |  |  |  |
| **C_mic_/C_org_** | p=0.235 | p=0.162 | p=0.634 | p=0.465 | p=0.183 | **p<0.001** |  |  |
| **qO_2_** | p=0.773 | **p=0.011** | p=0.450 | p=0.834 | **p=0.001** | p=0.872 | p=0.899 |  |
| **Ergosterol** | p=0.853 | p=0.658 | **p=0.026** | **p=0.007** | p=0.718 | **p<0.001** | **p=0.001** | p=0.098 |
|  |  |  |  |  |  |  |  |  |
| (c) | **C_R_** | **N_R_** | **C_C_** | **N_C_** | **C/N** | **C_mic_** | **C_mic_/C_org_** | **qO_2_** |
| **N_R_** | p=0.872 |  |  |  |  |  |  |  |
| **C_C_** | **p<0.001** | p=0.071 |  |  |  |  |  |  |
| **N_C_** | **p<0.001** | **p=0.026** | **p<0.001** |  |  |  |  |  |
| **C/N** | **p=0.014** | **p<0.001** | **p=0.001** | **p=0.002** |  |  |  |  |
| **C_mic_** | **p<0.001** | **p=0.011** | **p<0.001** | **p=0.001** | **p<0.001** |  |  |  |
| **C_mic_/C_org_** | **p<0.001** | **p=0.045** | **p<0.001** | **p<0.001** | **p<0.001** | **p<0.001** |  |  |
| **qO_2_** | **p<0.001** | p=0.202 | **p=0.014** | p=0.229 | **p=0.006** | **p<0.001** | **p<0.001** |  |
| **Ergosterol** | p=0.155 | p=0.865 | p=0.845 | p=0.374 | p=0.378 | **p=0.003** | **p=0.002** | **p=0.031** |
|  |  |  |  |  |  |  |  |  |
| (d) | **C_R_** | **N_R_** | **C_C_** | **N_C_** | **C/N** | **C_mic_** | **C_mic_/C_org_** |  |
| **N_R_** | **p=0.038** |  |  |  |  |  |  |  |
| **C_C_** | **p<0.001** | **p=0.002** |  |  |  |  |  |  |
| **N_C_** | **p<0.001** | **p=0.018** | **p<0.001** |  |  |  |  |  |
| **C/N** | **p<0.001** | **p<0.001** | **p<0.001** | **p=0.018** |  |  |  |  |
| **C_mic_** | p=0.556 | p=0.641 | p=0.607 | p=0.519 | p=0.959 |  |  |  |
| **C_mic_/C_org_** | p=0.486 | p=0.905 | p=0.658 | p=0.182 | p=0.584 | **p<0.001** |  |  |
| **qO_2_** | p=0.850 | p=0.661 | p=0.940 | p=0.275 | p=0.208 | **p=0.014** | **p=0.014** |  |
|  |  |  |  |  |  |  |  |  |
| (e) | **C_R_** | **N_R_** | **C_C_** | **N_C_** | **C/N** | **C_mic_** | **C_mic_/C_org_** |  |
| **N_R_** | p=0.657 |  |  |  |  |  |  |  |
| **C_C_** | **p<0.001** | **p=0.003** |  |  |  |  |  |  |
| **N_C_** | **p<0.001** | **p<0.001** | **p<0.001** |  |  |  |  |  |
| **C/N** | **p<0.001** | **p<0.001** | **p<0.001** | **p<0.001** |  |  |  |  |
| **C_mic_** | **p=0.007** | p=0.160 | **p=0.013** | **p=0.002** | **p=0.027** |  |  |  |
| **C_mic_/C_org_** | **p<0.001** | p=0.214 | **p=0.001** | **p<0.001** | **p=0.007** | **p<0.001** |  |  |
| **qO_2_** | **p=0.038** | p=0.346 | **p=0.019** | **p=0.015** | p=0.082 | **p<0.001** | **p<0.001** |  |
